# Editorial: Assistance personalization/customization for human locomotion tasks by using wearable lower-limb robotic devices

**DOI:** 10.3389/frobt.2024.1448100

**Published:** 2024-07-03

**Authors:** Qiang (Jason) Zhang, Xuefeng Bao, Zhao Guo, Ge Lv, Myunghee Kim

**Affiliations:** ^1^ Department of Mechanical Engineering, University of Alabama, Tuscaloosa, AL, United States; ^2^ Department of Chemical and Biological Engineering, University of Alabama, Tuscaloosa, AL, United States; ^3^ Department of Biomedical Engineering, University of Wisconsin–Milwaukee, Milwaukee, WI, United States; ^4^ School of Power and Mechanical Engineering, and Wuhan University Shenzhen Research Institute, Wuhan University, Wuhan, Hubei, China; ^5^ Department of Mechanical Engineering, Clemson University, Clemson, SC, United States; ^6^ Department of Mechanical and Industrial Engineering, University of Illinois Chicago, Chicago, IL, United States

**Keywords:** wearable robotics, human-in-the-loop, human-machine-interaction, locomotion assistance, optimal control

## Introduction

In recent years, the advancement of wearable lower-limb robotic devices has opened new avenues in the field of assistive technologies, particularly in enhancing human locomotion. These devices, often referred to as exoskeletons or robotic orthoses, are designed to support, enhance, or augment the movement capabilities of their users. This technology holds significant promise for individuals with mobility impairments, aging populations, and even healthy individuals in occupational settings where enhanced endurance or strength is beneficial.

The core objective of these robotic aids is to facilitate more natural and efficient movement patterns, thereby reducing the physical strain on the body and enabling longer periods of mobility. This is particularly crucial for rehabilitation purposes, where consistent and correct movement patterns can expedite recovery and potentially restore normal locomotor functions. The integration of customization and personalization in the design and functionality of these devices is critical, as it allows for adjustments tailored to the specific needs and conditions of each user. This approach not only improves the effectiveness of the technology but also enhances user comfort and satisfaction.

The personalization of assistance involves adjusting various parameters such as torque, timing of actuation, and kinematic profiles to match the user’s unique gait patterns and biomechanics. Customization can also extend to the software interfaces, where machine learning algorithms predict and adapt to the user’s preferred movement styles or changes in their physical condition over time. This level of personalization is achieved through sophisticated sensors and data analytics, which monitor the user’s movements and provide real-time feedback to the control systems of the wearable devices.

## Overview of the contents of the research topic structured

### Predicting the metabolic cost of exoskeleton-assisted squatting using foot pressure features and machine learning

Ramadurai et al. demonstrated the efficacy of using Center of Pressure (CoP) trajectory features to predict the metabolic cost of exoskeleton-assisted squatting, evidenced by a strong correlation between actual and predicted costs. Notably, the trajectory features corresponding to ankle inversion and eversion, labeled as xCoP, exhibited a significant positive correlation with metabolic expenditure. We observed that increased ankle eversion, which involves an outward rolling of the ankle, correlates with a higher metabolic cost. This biomechanical pattern is also associated with a heightened risk of chronic lower limb injuries, suggesting that xCoP trajectory features can serve as indicators of both metabolic cost and potential injury risk during squatting activities. The introduction of a CoP-based cost function in human-in-the-loop optimization presents multiple benefits. It not only reduces the time required for metabolic cost estimation but also mitigates injury risk and enhances overall user comfort. Crucially, this approach enables the application of human-in-the-loop optimization beyond laboratory settings, facilitating more practical and widespread use.

### Using human-in-the-loop optimization for guiding manual prosthesis adjustments: a proof-of-concept study

Senatore et al. employed a human-in-the-loop optimization algorithm to guide adjustments for optimizing a prosthetic simulator. The results indicate potential applicability for amputees, though numerous factors must be considered. Given that prosthetic components influence the load on the opposite limb, directly optimizing prosthesis parameters could be more clinically relevant for amputees. Additionally, because amputees depend on sensory feedback from their prostheses, focusing on optimizing a cost function that doesn't rely on physiological changes might prove more advantageous. Prosthetists typically consider both limbs when fitting and adjusting prostheses. Therefore, future research could explore a multi-objective optimization approach to assess the impact of varying multiple parameters on both limbs simultaneously. This would provide a broader understanding of the biomechanical interactions and enhance the effectiveness of prosthetic fittings.

### Swing-phase detection of locomotive mode transitions for smooth multi-functional robotic lower-limb prosthesis control

Haque et al. developed a new method called multi-dimensional dynamic time warping (mDTW) for recognizing different walking patterns and transitions in the swing phase of walking, aimed at improving robotic prostheses. This method helps the prosthesis anticipate and assist with actions that require more power in the following stance phase. Their study collected crucial gait data from multiple mechanical sensors to build an effective classifier. In creating the mDTW algorithm, we selected the six most informative sensor signals for input and enhanced the algorithm with a voting mechanism to maximize the use of collected data across different users. Their validation showed that the mDTW algorithm accurately identifies walking patterns or transitions within the first 30% of the gait cycle with 99.08% accuracy and an F1-score of 0.9730. Such early detection during the swing phase allows the prosthesis control system enough time to adjust its operation before the stance phase begins. Moreover, the algorithm is computationally efficient and easily personalized through individual user templates. This makes the mDTW intent recognizer a potential cornerstone for future prosthesis control systems, enhancing the usability of robotic prostheses for a broad amputee population.

### Lower limb biomechanics of fully trained exoskeleton users reveal complex mechanisms behind the reductions in energy cost with human-in-the-loop optimization

Poggensee and Collins investigated how different levels of plantarflexion assistance affect biomechanical responses. The primary changes in gait were seen at the assisted joint, which included an increased peak plantarflexion angle at toe-off, a reduction in peak biological ankle moment, and plantarflexor muscle activity, alongside an increase in biological ankle power. These changes corresponded to a decrease in overall body energy expenditure. The kinematics of the hip and knee remained largely unchanged, confirming findings from previous studies. However, these joints showed an increase in muscle activity. While joint work at these unassisted joints decreased with generic assistance, the overall reduction in metabolic cost suggests more complex interactions that this analysis alone cannot fully explain. These biomechanical insights could inform training protocols, musculoskeletal simulations, or the design of new devices. Further research is recommended, particularly with additional sensors to capture internal musculoskeletal dynamics, varied device controls, and diverse participant demographics.

## Conclusion

As emphasized in the above-mentioned articles, when we continue to explore the confluence of biomechanics, robotics, and artificial intelligence, the development of these personalized wearable devices for locomotion assistance emerges as a cornerstone of innovation. It not only promises to elevate the standard of assistive technology but also paves the way for greater independence and a better quality of life for those facing mobility challenges. The future of wearable technology in mobility assistance looks promising, with vast potential for further advancements that could redefine human-machine interaction for enhanced daily living and clinical outcomes.

